# Magnitude and associated factors of depression among people with hypertension in Addis Ababa, Ethiopia: a hospital based cross-sectional study

**DOI:** 10.1186/s12888-022-03972-6

**Published:** 2022-05-10

**Authors:** Yonatan Asmare, Ahmed Ali, Ayele Belachew

**Affiliations:** School of Public Health, College of Health Sciences, Addis Ababa University, Addis Ababa, Ethiopia

**Keywords:** Depression, Hypetension, Ethiopia

## Abstract

**Background:**

The burden of depression is higher among people with chronic illnesses like hypertension and this comorbid condition leads to poor adherence to treatment and failure of compliance to lifestyle modifications, which in turn, increases risk of cardiovascular complications and mortalities. Low income countries, Ethiopia included, suffer from paucity of information describing the burden of hypertension comorbid with depression, which demands studies to narrow this knowledge gap, such as this one.

**Methods:**

Institution based cross-sectional study was conducted in three randomly selected public hospitals in Addis Ababa. Through a systematic random sampling method, a total of 416 known hypertensive patients with follow up in hypertension clinics with in the study period enrolled in the study. Data were collected through structured questionnaire administered by trained interviewer, which latter cleaned, edited and entered in to epi-data version 3.1. Descriptive and bi-variable and binary logistic regression analysis were done using the statistical software, SPSS version 25. Depression was assessed through Hospital Anxiety and Depression Scale (HADs).

**Results:**

The prevalence of depression among hypertensive patients was found to be 37.8% [95% CI (33.4%-42.5%)]. The binary logistic regression model revealed that, female sex [AOR = 5.37, 95% CI (3.089–9.35)], being married [AOR = 0.25, 95% CI (0.08–0.78)], presence of chronic comorbid illnesses [AOR = 3.03, 95% CI (1.78–5.16)], uncontrolled blood pressure [AOR = 2.80, 95% CI (1.65–4.75)], duration of hypertension of 5–10 years [AOR = 3.17, 95% CI (1.61–6.23)] and more than 10 years [AOR = 5.81, 95% CI (2.90–11.65)], family history of depression [AOR = 4.53, 95% CI (2.37–8.66)] and current alcohol consumption [AOR = 1.77, 95% CI (1.02–3.07)] were significantly associated with depression among hypertensive patients.

**Conclusion:**

High proportion of depression was observed among hypertensive patients, and socio-demographic, clinical and behavioral characteristics were found to significantly influence the likelihood of occurrence. Health care providers should consider mental health status of hypertensive patients, and counsel for these factors.

## Background

Non-communicable diseases are the leading causes of morbidity and mortality in today’s world [1]. In 2016, non-communicable diseases were estimated to account for 71% of the 57 million global deaths. In addition to that, 78% of all non-communicable disease deaths occurred in low- and middle-income countries [2].

Mental disorders are common in all countries of the world and greatly affect socioeconomic development and growth [3]. Among the mental disorders, depression is a significant contributor to the global burden of disease and affects people in all communities across the world. Today, globally around 350 million people affected by depression with a 7% lifetime risk. By 2030 depression shall become the leading cause of disability worldwide [4].

Depression is a common mental disorder that presents with depressed mood, decreased energy, loss of interest, feelings of guilt or low self-worth, disturbed sleep or appetite, and poor concentration [5]. Moreover, Depression reduces individual’s productivity at work, school or daily activities of life, at severe stages, it can even lead to suicide [4].

Patients with chronic illnesses are at risk of developing mental illness. When psychological and psychosocial issues co-occur with physical illness, particularly with chronic diseases, that makes the diagnosis and management often difficult [6]. Differently from the general population, depression is consistently higher in people affected by chronic diseases [7]. Like patients with other chronic medical conditions, hypertensive patients experience many profound emotions which increase their risk for the development of mental health disorders, particularly anxiety and depression [8]. Even though studies are scarce in the area, a document from WHO shows higher prevalence of major depression in hypertensive patients [7].

Although the causal mechanism of depression and hypertension have not been proven yet, relationship of depressive symptoms with vascular diseases, such as hypertension and cardiovascular disease and increased risk of depressed patients to many illnesses related to vascular disease is known. However, so far there is no evidence to definitively state which condition occurs first [9].

Depression in hypertensive patients is associated with poorer health status, including lower quality of life [10], increased medical resources [11], lower rate of treatment compliance [12] and even increased mortality [13]. People with depression could suffer from lack of occupational and social role function and it is easier for hypertensive patients with depression to further develop depressive symptoms [14]. When uncontrolled blood pressure and poor adherence to antihypertensive medication is recognized among hypertensive patients, it is important to consider depression as a co-factor [15].

Comorbid mental disorders are often unrecognized and not always effectively treated [8]. Considering increasing prevalence and mortality of hypertension, there is a need for further research on psychological aspects of those diseases in Ethiopia. The prevalence and determinant factors of depression in hypertensive patients show inconsistent findings in studies from abroad [14]. In Ethiopia, depression among hypertensives is not well explored and fully understood. This study hence, aims to fill this knowledge gap.

## Methods

### Study area and period

The study was conducted in Addis Ababa, the Capital City of Ethiopia. There are ten public hospitals in Addis Ababa, which give chronic care, out of those, three hospitals were selected. The hospitals were St. Paul’s Hospital Millennium Medical College, Yekatit 12 Hospital Medical College and the All African Tuberculosis and Leprosy Rehabilitation and Training Hospital. The study was conducted between September 11, 2019 and November 17, 2020.

### Study design

A hospital based cross-sectional study design was employed.

### Study population

The study population included all hypertensive patients who were older than 18 years and under treatment for hypertension for at least six months in selected public hospitals hypertension follow up clinic during the data collection period. Critically ill patients, patients with severe psychiatric disorder and pregnant women who could have gestational hypertension were excluded.

### Study variables

The dependent variable was depression disorder, while independent were socio-demographic and economic characteristics (age, sex, marital status, monthly income, employment, level of education, residence), behavioral characteristics (alcohol consumption, cigarette smoking, physical activity) and psychosocial and clinical characteristics (comorbidities, blood pressure control, duration of hypertension, family history of hypertension and family history of depression).

### Sample size

Sample size was determined using a single population proportion formula with a 95% CI and 5% margin of error, assuming the prevalence of depression at 41.7% from a previous study [16]. Considering a 10% non-response rate, a total 416 hypertensive patients were involved in the study.

### Sampling procedures

Out of 10 public hospitals, that give hypertension follow-up services, three were randomly selected using a lottery method. The number of study units from each hospital were proportionally allocated from the three month’s patient visit load. Accordingly, 89, 65 and 262 cases were allocated to ALERT Hospital, St. Paul’s Hospital Millennium Medical College and Yekatit 12 Hospital Medical College respectively, and then individual cases were recruited by systematic random sampling procedure.

### Data collection procedures

Data were collected through structured questionnaire through face to face interview by four trained BSc nurses. The instrument has three sections; the first section assessed socio-demographic and behavioral characteristics which was modified from the World Health Organization instrument for stepwise surveillance (WHO STEPS) of chronic disease risk factors [17], while the second section assessed clinical characteristics, and the third section assessed depression using the Hospital Anxiety and Depression Scale (HADS) [18]. The score classified as normal or no depression (0–7), mild depression [8–10] and marked depression [11–21]. The participant’s blood pressure and comorbidities were taken from their medical records. The tool has been validated earlier in Ethiopia [20].

### Data Analysis procedures

Data were checked for completeness, cleaned, edited, coded and entered to epi-data 3.1 version and exported and analyzed using SPSS version 25, and descriptively analyzed and displayed by frequencies, means, median and proportions. Bivariate analysis was calculated to examine association of independent variables with the dependent variable. Variables with P-value < 0.25 were entered to binary logistic regression model to examine the association of each predictor variable in the outcome variable and control the effects of confounding variables for depression, using odds ratios, with 95% confidence interval. Independent variables with P-value of < 0.05 were considered as having statistical significant association with the outcome variable.

### Data quality assurance

A Properly designed data collection tool was prepared. The questionnaire was assessed for simplicity and clarity. The English version of the questionnaire was translated in to the local language, (Amharic) and translated back to English, to check for consistency. The original and translated questionnaires were compared and the discrepancies were reviewed and resolved accordingly. Pretest was made in 5% (21 hypertensive patients) of the target population at Ras Desta Hospital and necessary modifications were made. The data collected were reviewed and checked for completeness and relevance by supervisor and principal investigator each day.

### Operational definitions

#### Hypertension

Is defined as a rise in blood pressure when systolic blood pressure ≥ 140 mm Hg and/or diastolic blood pressure ≥ 90 mm Hg [21].

#### Depression

Score of greater than or equal to 8 by the HAD depression sub-scale.

Controlled blood pressure is systolic < 140 and diastolic < 90 and uncontrolled blood pressure is systolic ≥ 140 or diastolic ≥ 90) [22].

Adults were categorized as current smoker when they were using cigarette in the past one month and when individuals smoked at least once in their life time they were categorized as ever smoker. Adults were categorized as current drinker when they were using alcohol in the past one month and when individuals drink alcoholic beverages at least once in their life time they were categorized as lifetime drinker [17].

#### Physically active

Those who were doing moderate to vigorous activities at work or leisure time for at least 30 min, for 4 or more days in a week [23], in accordance with WHO typical physical activities [17].

#### Comorbid illnesses

Arthritis, HIV/AIDS, Leprosy, asthma, any other chronic lung diseases (COPD or emphysema), stroke, heart attack, heart disease, chronic kidney disease, chronic liver disease, diabetes, thyroid disease, neurological problems (seizure, epilepsy, parkinson’s), cancer.

## Results

### Socio-economic, demographic, clinical and behavioral characteristics of respondents

#### Socio-economic and demographic characteristics of respondents

Complete data was obtained and analyzed from 407 participants (response rate of 97.8%), of which 219 (53.8%) were males, and the mean age was 49.47 years (standard deviation of 11.46), the majority (33.7%) were in the age group 48–57 years. More than two third (67.8%) were married and one third (40.8%) attended college level education (Table 1). The monthly median average income was 74.5 USD (inter-quartile range of 44.7 USD).

**Table 1:** Socio-economic and demographic characteristics of hypertensive patients attending public hospitals in Addis Ababa, 2020 G.C

Variables		Frequency (*n* = 405)	Percent (%)
Sex	Male	219	53.8
	Female	188	46.2
Age	18–27 years	10	2.5
	28–37 years	55	13.5
	38–47 years	117	28.7
	48–57 years	137	33.7
	58–67 years	65	16
	68 and above years	23	5.7
Educational status	Unable to read and write	56	13.8
	Primary education	94	23.1
	Secondary and preparatory education	91	22.4
	College and above	166	40.8
Employment status	Non-governmental employee	26	6.4
	Government employee	142	34.9
	Self-employed	29	7.1
	Non-paid job	15	3.7
	Homemaker	53	13
	Retired	74	18.2
	Unemployed (able to work)	28	6.9
	Unemployed (unable to work)	40	9.8
Marital status	Married	276	67.8
	Single	31	7.6
	Widowed	28	6.9
	Divorced	72	17.7
Residence	Urban	403	99
	Rural	4	1
Monthly average income	< 30 USD	95	23.3
	30–107 USD	272	66.8
	108–322 USD	28	6.9
	≥ 323	12	2.9

#### Clinical and individual characteristics of respondents

As it is shown in Table 2, at least one or more comorbid conditions were reported by 168 (41.3%). Duration of hypertension in 124 (30.5%) of the participants was greater than 10 years, and their blood pressure was under control in more than half (57.5%). Family history of hypertension was reported only by 52 (12.8%), while family history of depression by 80 (19.7%). Furthermore, history of smoking and alcohol drinking was reported by 9.8% and 70.5% respectively, while physical activity by 14.5% (Table 2).

**Table 2:** Clinical, behavioral and individual characteristics of hypertensive patients attending public hospitals in Addis Ababa, 2020 G.C

Variables	Category	Frequency (*n* = 405)	Percent (%)
Comorbid illness	Yes	168	(41.3)
	No	239	(58.7)
Blood pressure control	Yes	234	(57.5)
	No	173	(42.5)
Duration of Hypertension	< 5 years	131	(32.2)
	5–10 years	152	(37.3)
	> 10 years	124	(30.5)
Family history of hypertension	Yes	52	(12.8)
	No	355	(87.2)
Family history of depression	Yes	80	(19.7)
	No	327	(80.3)
Ever smoke	Yes	40	(9.8)
	No	367	(90.2)
Current smoke	Yes	17	(4.2)
	No	390	(95.8)
Ever alcohol	Yes	287	(70.5)
	No	120	(29.5)
Current alcohol	Yes	185	(45.5)
	No	222	(54.5)
Physical activity	Yes	59	(14.5)
	No	348	(85.5)

#### Prevalence of depression among hypertensive patients

The prevalence of depression among hypertensive patients was found to be 37.8% [95% CI (33.4%-42.5%)], 24.6% of them were mildly depressed and 13.2% were markedly depressed.

#### Factors associated with depression

Binary logistic regression analysis revealed that females were 5.4 times more likely to have depression compared to males [AOR = 5.37, 95% CI (3.089–9.35)]. Married subjects had 75% reduced odds of having depression compared to those who were single [AOR = 0.25, 95% CI (0.08–0.78)]. Those who had comorbid illnesses were 3 times more likely to have depression compared to those with no any comorbid illness [AOR = 3.03, 95% CI (1.78–5.16)], those with uncontrolled blood pressure are 2.8 times more likely to have depression compared to those with controlled blood pressure [AOR = 2.80, 95% CI (1.65–4.75)], subjects with duration of hypertension 5 to 10 years are 3.2 times [AOR = 3.17, 95% CI (1.61–6.23)] and more than 10 years are 5.8 times [AOR = 5.81, 95% CI (2.90–11.65)] more likely to have depression compared to patients with less than 5 years of hypertension duration. Subjects with positive family history of depression are 4.5 times more likely to have depression compared to their counterparts [AOR = 4.53, 95% CI (2.37–8.66)]. Current alcohol drinkers were 1.8 times more likely to have depression compared to those who are not alcohol drinkers currently [AOR = 1.77, 95% CI (1.02–3.07)] (Table 3).

**Table 3:** Factors associated with depression among hypertensive patients attending public hospitals in Addis Ababa, 2020 G.C

Variables	Depression N (%)		COR(CI)	AOR(CI)	*P* value
	**Yes**	**No**			
**Sex**					
Male	56(25.6)	163(74.4)	1	1	
Female	98(52.1)	90(47.9)	3.17(2.09–4.81)	5.37(3.08–9.35)	0.000***
**Marital status**					
Married	62(22.5)	214(77.5)	0.40(0.17–0.99)	0.25(0.08–0.78)	0.017*
Divorced	26(36.1)	46(63.9)	0.78(0.47–1.38)	0.94(0.48–1.83)	0.860
Widowed	7(25)	21(75)	0.46(0.20–1.15)	0.42(0.12–1.41)	0.159
Single	13(41.9)	18(58.1)	1	1	
**Monthly average income**					
< 30 USD	53(55.8)	42(44.2)	1.77(0.52–5.97)	0.80(0.17–3.80)	0.779
30–107 USD	81(29.8)	191(70.2)	0.59(0.18–1.93)	0.32(0.07–1.38)	0.126
108–322 USD	15(53.6)	13(46.4)	1.62(0.41–6.34)	0.57(0.10–3.24)	0.528
≥ 323 USD	5(41.7)	7(58.3)	1	1	
**Comorbid illness**					
Yes	88(52.4)	80(47.6)	2.88(1.90–4.37)	3.03(1.78–5.16)	0.000***
No	66(27.6)	173(72.4)	1	1	
**Blood pressure control**					
Yes	60(25.6)	174(74.4)	1	1	
No	94(54.3)	79(45.7)	3.45(2.27–5.25)	2.80(1.65–4.75)	0.000***
**Duration of Hypertension**					
> 10 years	66(53.2)	58(46.8)	4.83(2.75–8.45)	5.81(2.90–11.65)	0.000***
5–10 years	63(41.4)	89(58.6)	3.00(1.75–5.16)	3.17(1.61–6.23)	0.001**
< 5 years	25(19.1)	106(80.9)	1	1	
**Family history of depression**					
Yes	53(66.3)	27(33.7)	4.39(2.61–7.38)	4.53(2.37–8.66)	0.000***
No	101(30.9)	226(69.1)	1	1	
**Current alcohol**					
Yes	77(41.6)	108(58.4)	1.34(0.90–2.00)	1.77(1.02–3.07)	0.041*
No	77(34.7)	145(65.3)	1	1	

^*^Statistically significant at *p*-value < 0.05, ** statistically significant at *p*-value < 0.01, *** statistically significant at *p*-value < 0.001, COR = crude odds ratio at 95% confidence interval; *AOR* adjusted odds ratio at 95% confidence interval

## Discussion

This study revealed the prevalence of depression among hypertensive patients to be 37.8%, the finding is in line with studies done in Ghana 41.7% [16], Pakistan 40.1% [24] and India 41% [25]. On the other hand, the current finding is lower than a study conducted in Afghanistan, which found prevalence of depression among hypertensives to be 58.1% [26]. As suggested by the researchers, the reason for higher magnitude could be the ongoing conflict in different parts of the country. Similarly, higher prevalence of depression observed in a study from Saudi Arabia 48.7% [27] and Bosnia and Herzegovina 46% [28]. Ebtesam B found the highest prevalence of depression which is in two third (66.7%) of hypertensive subjects [29]. On the contrary, lower prevalence observed in few studies a study in Hawassa, Southern Ethiopia [30], Ghana [31] and Nigeria [16]. The variations might be due to difference in environmental factors, genetical factors, sample size, method of assessment of depression and data collection tool [14]. Higher magnitude of depression in this study could be attributed to COVID-19 pandemic at the time of data collection [32].

According to this study, hypertensive females are at 5.4 times increased chance to be depressed. This is higher than a study in South Africa 3.5 times [33] and much higher than a study in Hawassa, Southern Ethiopia, 2.6 times increased risk [30]. Similarly, majority of studies in the literature suggests the same findings [24, 25, 27–29]. Sociocultural variation could be the reason for the difference. In addition to this, higher magnitude of depression in females could rise from changes in sex hormone and influences in females related to social norms and gender issues, which is parents restrictive behavior towards their daughter than their sons affects their daughters senses of self-control and self-esteem and make them vulnerable to depression. Sexual and domestic violence could also contribute for the higher risk in females [34].

With respect to marital status, hypertensive subjects who were married have 75% reduced chance of being depressed. This finding is consistent with prior reports in the literature [25, 28]. Differently from this, studies in Ethiopia [30], South Africa [33], Pakistan [24], Afghanistan [26] and Ghana [16] found no association. The reason for reduced risk of depression among married could be being settled, sharing the increasing burden of living cost and retaining positive health behaviors [35].

In terms of comorbid illnesses, our study revealed that hypertensive patients with comorbid illnesses are 3 times more likely to be depressed. An Afghanistanian study reported that hypertensive subjects with comorbid diabetes are 22.7 times more likely to have depression [26]. Similar to this, Vishnu G and et al. found higher magnitude of depression among hypertensives with comorbidities [25]. A plausible explanation for that could be, the sadness and consistent hardship the comorbid illnesses bring together [7].

In respect to blood pressure control, this study also found that hypertensive subjects who had uncontrolled blood pressure are 2.8 times more likely to be depressed. This finding is in accordance with a study in Saudi Arabia [29]. It could be because of poor adherence of depressed subjects to their medications resulting in poor blood pressure [7].

Regarding duration of hypertension, this study showed that those with increased duration of hypertension likelihood of being depressed increased. Subjects with duration of hypertension 5 to10 years and more than 10 years have 3.2 and 5.8 times increased chance of being depressed respectively. This finding is comparable to a previous study which was conducted in Pakistan [25]. It is also supported by Kosana S et al. report, depression is significantly more expressed in hypertensives with prolonged duration of the disease [28].

With respect to family history of depression, this study found that hypertensive participants with family history of depression are 4.5 times more likely to have depression. This association is demonstrated by a study in Hawassa, Southern Ethiopia, where hypertensive subjects with positive family history had 7 times higher chance of being depressed [30]. Similarity in genetical factors could be the reason [14].

Besides, our study also showed that hypertensive subjects who drink alcohol have 1.8 times increased chance of being depressed. This finding is comparable with a study conducted in South Africa, with 1.9 times higher risk of being depressed [33]. A review by Tesera B revealed higher odd of depression among substance abusers than their counterparts [34]. The possible explanation could be influence of health effect, social problem, economic and productivity loss associated with alcohol increase the risk of depression [36].

## Conclusion

High prevalence of depression was observed among hypertensive patients attending the selected public hospitals in Addis Ababa. Depression is significantly associated with female gender, being married, presence of comorbid illnesses, uncontrolled blood pressure, longer duration of hypertension, family history of depression and current alcohol drinking. The study recommends that hypertension follow-up clinics should establish a mechanism of screening service for mental health, in particular for depression. Moreover, further nation-wide community-based researches are needed to assure generalizability of the findings.

## Data Availability

The datasets used and/or analysed during the current study are available from the corresponding author on reasonable request.

## Abbreviations

AOR: Adjusted Odds Ratio
ALERT: All African Tuberculosis and Leprosy Rehabilitation and Training Hospital
COR: Crude Odds Ratio
HADS: Hospital Anxiety and Depression Scale
SPSS: Statistical Package for Social Science
USD: United States Dollar
